# Preparation of Filter Paper from Bamboo and Investigating the Effect of Additives

**DOI:** 10.3390/ma17091977

**Published:** 2024-04-24

**Authors:** Zahra Kazemi Karchangi, Noureddin Nazarnezhad, Jalel Labidi, Seyed Hassan Sharifi

**Affiliations:** 1 Wood and Paper Science Department, Faculity of Natural Resources, Sari Agricultural Sciences and Natural Resource University, Sari 4818168984, Mazandaran, Iran; zkazemi71@yahoo.com (Z.K.K.); h.p.sharifi@gmail.com (S.H.S.); 2 Chemical and Environmental Engineering Department, University of the Basque Country UPV/EHU, Plaza Europa, 1, 20018 Donostia-San Sebastián, Spain

**Keywords:** filter paper, bamboo, soda AQ, cationic starch, polyvinyl alcohol

## Abstract

As air pollution escalates, the need for air filters increases. It is better that the filters used be based on natural fibers, such as non-wood fibers, which cause low damage to the environment. However, the short fiber lengths, low apparent densities, and high volumes of non-wood materials can make it challenging to prepare filter paper with the required mechanical and physical properties. In that context, this study focused on utilizing bamboo fibers to fabricate filter paper by employing the anthraquinone soda pulping method. The pulp underwent bleaching and oxidation processes, with the incorporation of cationic starch (CS) and polyvinyl alcohol (PVA) to enhance resistance properties, resulting in the creation of handmade filter papers. The findings revealed that the tear, burst, and tensile strength of filter paper increased with the oxidation and addition of CS and PVA. Air permeability increased with addition of PVA and combination of CS and PVA. FTIR demonstrated the conversion of hydroxyl groups in cellulose chains to carboxyl groups due to oxidation. SEM images illustrated alterations in the fiber structure post-oxidation treatment, with CS reducing pores while PVA and the CS-PVA combination enlarged pore size and enhanced porosity. The BET surface area surface area expanded with oxidation and the addition of the CS-PVA blend, indicating heightened filter paper porosity. Notably, the combined inclusion of CS and PVA not only augmented mechanical strength but also increased porosity while maintaining pore size.

## 1. Introduction

As air pollution rises, there is a growing focus on filtration devices. The main components of air pollutants are suspended solid particles, liquid aerosol, biological droplets, metal smoke, gas, and vapor [1]. Air filter media are usually made of petroleum-based synthetic fiber polymers such as polypropylene, polyester, polyamide, and glass fibers. However, synthetic polymer fibers in air filters exhibit several drawbacks—they are non-recyclable, non-degradable, and involve complex production processes emitting greenhouse gases. In contrast, natural fibers present notable advantages as they occur naturally and are readily accessible. Cellulose fibers play a pivotal role in air filtration by effectively absorbing air pollutants. The production of air filters demands a reliable raw material source, and one promising alternative to synthetic polymers is the utilization of natural plant fibers [2]. Softwood fibers are better for filter preparation due to their longer fiber lengths and greater fibrillation [3]. However, in temperate regions where softwood is scarce, non-wood plants, particularly bamboo, offer a viable alternative due to their fiber lengths, which can rival those of woody plants [4]. The production of filter paper from non-wood sources presents numerous advantages, including simplified pulping processes, generation of high-quality bleached pulp, and suitability for crafting specialized papers [5].

Non-wood resources like bamboo offer superior technical suitability for paper production. Bamboo is a member of the grass family, which belongs to the family of Gramineae and sub-family of Bambusoidae and is distributed in subtropical and tropical areas. It presents several advantages over wood, including a shorter growth cycle, self-reproduction, and minimal maintenance and regeneration costs [6,7]. Globally, there are approximately 75 genera and 1250 species of bamboo in the world, which grow very fast and reach maturity within 5 years. The amount of D-cellulose in bamboo is about 40–50%, which is comparable to the amount of D-cellulose in hardwood and softwood [8]. 

When compared to non-woody fibers such as bagasse, reed, or wheat straw, bamboo’s exceptional qualities like long fiber length and high length-to-diameter ratio make it the preferred choice for paper production [9]. However, it is important to note that bamboo fibers have a shorter length compared to synthetic polymer fibers and softwoods [10]. Therefore, in order to increase the connections between the fibers and improve the mechanical resistance of the produced filter, it is necessary to use special treatments such as cross-linkers and additives [11]. In a study conducted by Peterus et al. [12], it was found that the addition of cationic starch to bamboo pulp increases structural bonding and is effective in improving the structure and mechanical properties of bamboo paper.

In the pulping process, it is better to use eco-friendly methods to prevent environmental damage. While many pulp and paper industries utilize the kraft method, the soda pulping method is known to cause less pollution. Additionally, adding AQ in the pulping process accelerates the degradation of lignin, increases the optical and physical properties, and maintains the performance of the pulp [13]. Studies indicate that when AQ is combined with a surfactant, compared to kraft pulp, the yield of unbleached pulp and pulp viscosity increase by 1.7–2% and 1.1–1.8 CP, respectively [4]. These air filters can be used in air purification from pollutants and dust. Accordingly, the goal of this research was to produce filter paper using the soda pulping method on environmentally friendly fibers instead of synthetic fibers and strengthen it with degradable CS and PVA additives.

## 2. Materials and Methods

The raw material used for this study was bamboo stalk of the species *Bambusa vulgaris*, which was collected from the garden of the Sari Natural Resources faculty (Sari, Mazandaran, Iran). Cationic starch with 0.035 degrees of substitution (DS) was obtained from the Mazandaran wood and paper mill (Sari, Mazandaran, Iran). Other chemicals used in this study were received from E-Merck, Darmstadt, Germany.

### 2.1. Preparation of Bamboo Chips and Fibers

First, random sampling was performed from bamboo stalks. Internodes were separated from nodes and chopped to the length of 3–4 cm with the same dimensions.

### 2.2. Pulping Conditions 

Pulping was carried out using 25% sodium hydroxide and a ratio of chemical to wood chips of 5:1 over 2 h and at temperatures of 175 and 185 °C. Pulping under different amounts of anthraquinone (AQ) (0, 0.1, and 0.2%) was investigated and the results were compared. After digestion, the pulp was washed till free from residual chemicals, and the pulp yield was measured [14].

### 2.3. Bleaching

Unbleached pulp was bleached by sequence DED. In this sequence, D represents chlorine dioxide and E represents alkaline extraction with NaOH. Chlorine dioxide consumption was based on Cl_2_ activity in the first stage, and 50% of this amount was used for the third stage of bleaching. The amount of profit in the second stage was considered equal to 0.6 of the consumption of chlorine dioxide in the first stage. The pulp consistency was 10% and the temperature was 70 °C in all stages [15]. 

### 2.4. Oxidation Treatment

In the initial phase, 6 g of pulp (based on dry weight) with a 3% consistency was subjected to chelation with 0.5% diethylenetriaminepentaacetic (DTPA) at a temperature of 60 °C for 30 min to eliminate heavy metal ions. Subsequently, the pulp was washed with distilled water.

In the subsequent stage, specific quantities of 3% sodium silicate and 3% hydrogen peroxide were added to the pulp, along with a sodium hydroxide-to-hydrogen peroxide ratio of 0.8. The pulp, with a consistency of 10%, was placed in a water bath set at 70 °C for 90 min. Following this step, the pulp was washed again with distilled water [16].

### 2.5. Additives

We used two additives, cationic starch (CS) and polyvinyl alcohol (PVA), which were prepared first. Cationic starch with a concentration of 0.2% was heated at 70 °C. The solution was kept at this temperature for 30 min. To prepare polyvinyl alcohol, 4 g of the solution was heated in 100 mL of water for 30 min at 80 °C. According to previous studies, 2% cationic starch and 3% polyvinyl alcohol were added to the suspension and stirred for 10 min. The time intervals for adding the additives and their retention times were the same for all treatments. Table 1 shows the different samples prepared.

### 2.6. Filter Paper Making

Handmade filter papers with a base weight of 20 g/m^2^ were made without using a press or dryer. Subsequently, the filter papers were kept overnight in an environment with conditions according to standard TAPPI T 402 [17] before testing. 

### 2.7. Testing Methods

The chemical compositions of the bamboo stalks, including alpha-cellulose, lignin, and ash, were analyzed according to Tappi T 203 [18], Tappi T 222 [19], and Tappi T 267 [20]. According to Franklin’s [13] method, bamboo fiber was prepared and the dimensions were measured with an optical microscope. The yield and kappa number of the pulp were determined according to Tappi T 210 [21] and Tappi T 236 [22]. The tensile index, burst index, and tear index of handsheet filters were measured according to Tappi T 494 [23], Tappi T 403 [24], and Tappi T 414 [25], respectively. Brightness and air permeance determinations of handsheet filters were also conducted in accordance with Tappi T 272 [26] and UNE-ISO 5636-3 [27], respectively. The network structure of the filters was captured using scanning electron microscopy (SEM) (Quant 250FEG, Hillsboro, OR, USA), and pore analysis was performed using ImageJ software (version 1.46r). Fourier transform infrared (FTIR) spectra were recorded using an FTIR spectrometer (NICOLET MODEL NEXUS 670, Madison, WI, USA) from 400 to 4000 cm^−1^. The specific surface areas of filter papers were determined by N2 adsorption at 77 K (using a Micromeritics ASAP 2420, Norcross, GA, USA) using the BET (Brunauer–Emmett–Teller) method. A one-way analysis of variance (ANOVA) statistical test was performed utilizing SPSS 18.0 software. Significant difference values were obtained by Duncan’s multiple range test. The results were expressed as the average and standard deviation. *p*-values < 0.05 were considered statistically significant.

## 3. Results and Discussion

### 3.1. Chemical Composition and Fiber Dimensions

The chemical composition analysis of bamboo stalks revealed that the mass percentages of cellulose, lignin, and extractives were 50, 25.7, and 0.75 wt%, respectively. These findings underscored the abundant cellulose content inherent in bamboo. The results of measuring the dimensions of fibers showed that the average length and diameter of the fibers of the studied samples were 2 mm and 15 microns, respectively. These dimensions positioned bamboo fibers between those of hardwoods (ranging from 0.7 to 3.0 mm) and softwoods (ranging from 2.7 to 4.6 mm) [28]. Consequently, it was anticipated that the produced filter resistances would be lower than those of softwoods and higher than those of hardwoods. It seems that the combination of a high growth rate, substantial cellulose content, and suitable fiber size renders bamboo an ideal non-wood material for various applications, including filter papers. 

### 3.2. Pulp Yield and Kappa Number

The overall yields of soda pulping were calculated with different amounts of AQ, as shown in Table 2. At a temperature of 185 °C, without using AQ, the yield of the pulp was 28%. Introducing 0.1% AQ increased the efficiency to 31%, and with 0.2% AQ, the yield of the pulp rose to 35%. 

Similarly, at a temperature of 175 °C, the total efficiency of pulp production was observed to increase with the addition of AQ. Without AQ, the yield was 35%, whereas with 0.1% AQ, it reached 38%, and with 0.2% AQ, the yield of the pulp further increased to 42%.

The best pulp yield was 42%, which was obtained at 175 °C temperature and 0.2% AQ. In soda pulping, a higher cooking temperature has a negative effect on pulp yield and leads to a lower pulp yield [29]. Also, increasing the amount of AQ increases the total yield of the pulp [13]. The results demonstrated that the responses of yield and kappa number were statistically significant.

In Table 2, kappa numbers and lignin percentages are provided. It can be observed that with increases in AQ addition of 0% to 0.2%, both kappa numbers and lignin percentages decreased, from 26.10 to 20.54 and from 46.46 to 17.5, respectively. This reduction can be attributed to the addition of AQ, which accelerates the alkaline peeling reaction through an oxidation–reduction mechanism. Consequently, the pulp yield increases while the kappa number decreases [30]. Given that the pulp prepared at 175 °C with 0.2% AQ exhibited the highest total yield and the lowest kappa number and lignin percentage, it was chosen for the subsequent steps in preparing filter paper.

### 3.3. Brightness

The DED sequence was used to achieve the desired brightness level. Prior to the bleaching process, the brightness was 25%. After the D1 stage, the brightness percentage increased to 65%, and after the D2 stage, it reached a final brightness of 85%.

Due to the low kappa number of the pulp, it achieved a high level of brightness with the DED sequence. This can be attributed to the reduction in light-absorbing chromophore groups, resulting in a lower light absorption coefficient and an increase in the scattering coefficient. These outcomes align with the findings reported by Resalati et al. [31].

### 3.4. Tear Strength 

**Figure 1:**
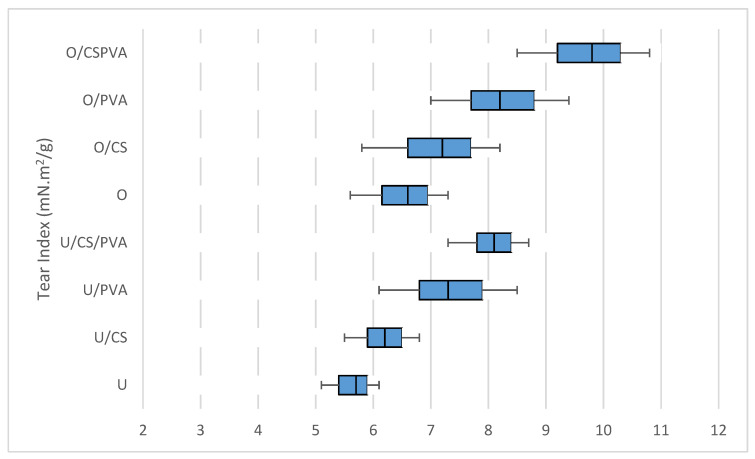
Effect of addition of cationic starch and PVA on the tear indexes of oxidized and unoxidized filter paper.

The tear index results of filter papers made from oxidized and unoxidized pulp, without additives and with additives, are shown in Figure 1 The average tear indexes for U, U/SC, U/PVA, and U/SC/PVA are 5.75, 6.3, 7.7, and 8.3 mN·m^2^/g, while for O, O/SC, O/PVA, and O/SC/PVA, they are 6.5, 7, 8.3, and 9.7 mN·m^2^/g, respectively. The tear index in oxidized filter papers is higher than that in unoxidized filter papers. Among the additives, PVA increased the tear resistance index more than cationic starch. By adding both additives, the highest tear index of this resistance was obtained. 

The tear resistance of filter paper depends on several factors, including the total number of fibers participating in the tear of the sheet, the lengths of the fibers, the number of connections, and the strengths of the bonds between the fibers [32]. An increase in tear resistance can be due to the formation of a cross-linked network in the PVA matrix [33]. 

### 3.5. Burst and Tensile Index

**Figure 2:**
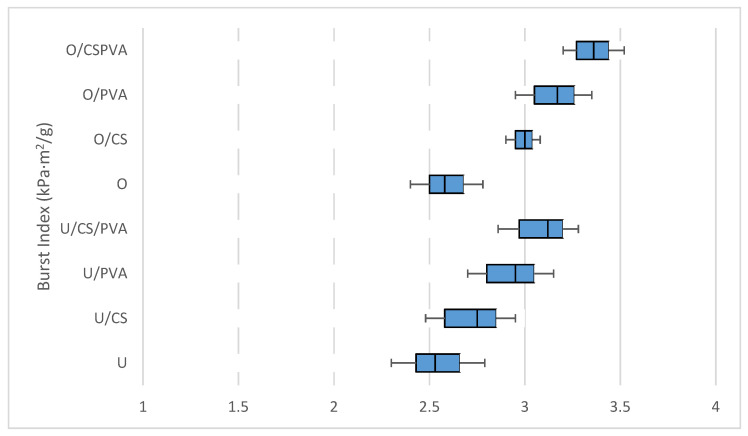
Effect of addition of cationic starch and PVA on the burst indexes of oxidized and unoxidized filter paper.

**Figure 3:**
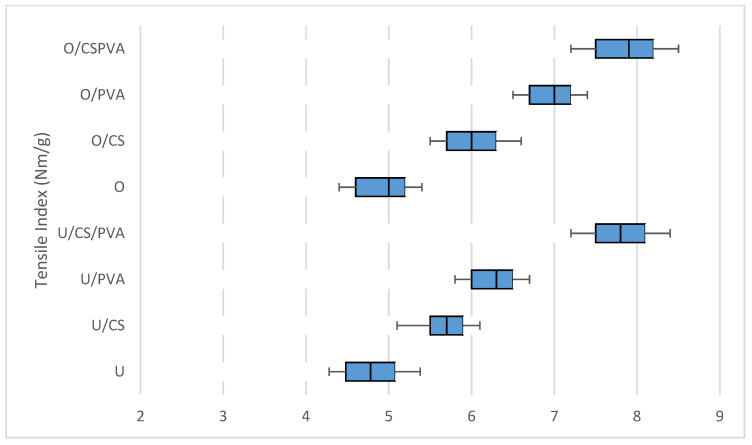
Effect of addition of cationic starch and PVA on the tensile indexes of oxidized and unoxidized filter paper.

Another important mechanical feature of filter paper is the burst resistance, which is a measure of the overall strength of the filter paper. The effects of individual and combined additions of CS and PVA on the burst index of filter paper are presented in Figure 2. It shows that the average burst indexes of U, U/SC, U/PVA, and U/SC/PVA were 2.5, 2.75, 3, and 3.12 kPa·m^2^/g, and those of the oxidized samples for O, O/SC, O/PVA, and O/SC/PVA were 2.6, 2.9, 3.2, and 3.4 kPa·m^2^/g respectively. Furthermore, tensile strength is related to burst resistance, and so the tensile strength of filter paper is a useful feature in the installation process of a filtration system [2]. Figure 3 shows the effects of individual and combined additions of CS and PVA on the tensile indexes of filter paper. The tensile indexes of U, U/SC, U/PVA, and U/SC/PVA were 4.3, 5.8, 6.3, and 7.3, Nm/g, while for O, O/SC, O/PVA, and O/SC/PVA, they were 4.6, 6.5, 7, and 7.8 Nm/g, respectively. These results showed that the burst and tensile index in oxidized filter papers was higher than that in unoxidized filter papers. Like the tear index, this resistance increased more with the addition of PVA than CS, and the combined addition created the best burst and tensile index in the manufactured filter papers.

According to previous studies, the increase in hydrogen bonds between fibers created by carboxyl groups in oxidized pulps increases the tensile and burst index [34]. The tensile and burst index of filter paper with the addition of CS increased compared to the control sample thanks to promoting bonding between the fibers [32]. Since PVA has a greater number of OH groups, which creates more strength and increases the tensile and burst index [35], it can be inferred that both CS and PVA directly contribute to the mechanical resistance of the prepared filter paper.

### 3.6. Air Permeability Measurement

Air permeability is an important property affecting the permeability of filter media. The air permeability of the filter paper determines the porosity and fluidity of the gas molecules that pass through the filter paper [36]. The results obtained from the air permeability tests appear in Table 3, indicating that the amounts of air permeance in the prepared filter papers were similar. Air permeability slightly increased in oxidized filter papers compared to the unoxidized filter paper, excluding O/PVA. This anomaly in O/PVA could be attributed to a potential increase in the cross-linking of fibers. According to Kan et al. [37], the dissolution of PVA increases the number of chemical bonds of oxide fibers.

Filter papers with CS added (U/CS and O/CS) exhibited a slightly decreased air permeability compared to their respective counterparts without CS, with values of 77.3 and 78.4 μm/Pa·s for U/CS and O/CS, respectively. Conversely, the addition of PVA resulted in slightly increased air permeability, reaching 80 and 79.4 μm/Pa·s in U/PVA and O/PVA, respectively. Remarkably, the combined addition of CS and PVA produced the highest air permeability among the manufactured filter papers, with a value of 83.8 μm/Pa·s for O/CS/PVA, signifying a delicate balance achievable through additive combinations. The difference between the samples’ averages for air permeability was statistically significant.

The addition of cationic starch (CS) may lead to fibers clustering together during drying, resulting in denser structures and decreased air permeability [38]. Consistent with the findings of Rice et al. [39], an increase in air permeance indicates heightened filter paper porosity. The addition of CS strengthens fiber bonds, increasing resistance to air permeance and subsequently reducing air permeability. These results are in line with the findings of Chen et al. [9], who utilized polyvinyl alcohol (PVA) in cellulose fiber composition, effectively increasing porosity and improving filtration efficiency and air permeability. In a filter containing both PVA and CS, the synergy of these cross-linkers creates proper connections between fibers without causing them to cluster too closely together. This balanced approach increases resistance and expands porosity, resulting in enhanced filter performance.

### 3.7. Scanning Electron Microscopy (SEM)

**Figure 4:**
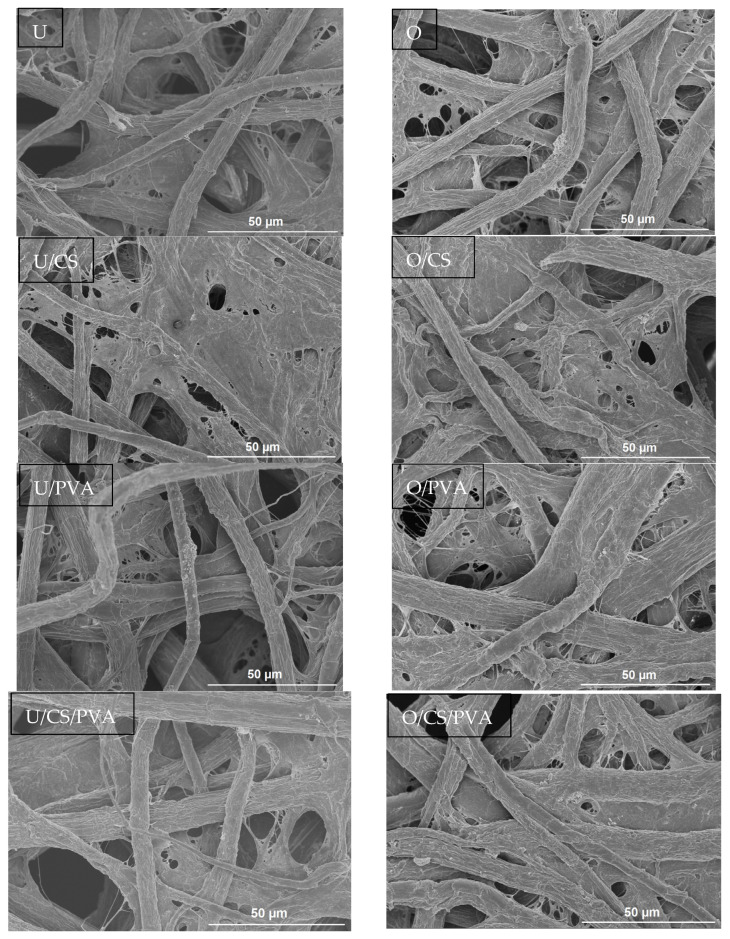
SEM images of prepared filter papers (magnification = 50×).

SEM images were used to analyze the morphological structures of filter papers. Figure 4 illustrates the SEM images of U, U/SC, U/PVA, U/SC/PVA, O, O/SC, O/PVA, and O/SC/PVA. Overall, the structure of oxidized fibers has changed compared to unoxidized fibers and different additives have affected the number and size of pores. Upon adding CS, numerous pores have been obstructed, as indicated by the circled region in Figure 4. This has resulted in fewer pores being formed compared to the control sample. Conversely, U/PVA and O/PVA filter papers exhibit an increase in both pore size and quantity. However, in O/PVA filter paper, the pores are smaller and less numerous compared to U/PV. The more porous structure formed in filter papers containing PVA may be attributed to its ability to create a spatial barrier during the cross-linking process [40]. Filter papers utilizing the combination of CS and PVA display pores with more homogeneity, exhibiting a porous microstructure conducive to good filtration performance.

Porosity and pore size were measured using ImageJ, and the values obtained for each sample are presented in Table 4. Generally, the unoxidized filter papers presented a larger average pore size and slightly lower porosity, while oxidized paper, exhibiting a smaller average pore size, managed to maintain a porosity level that was not significantly lower than that of its unoxidized counterpart. The addition of cationic starch led to reduced pore sizes and corresponding decreases in porosity in both unoxidized and oxidized samples. Specifically, in the unoxidized category, filter paper containing polyvinyl alcohol (U/PVA) displayed a moderate average pore size of 3.56 µm and a corresponding porosity of 3.39%. In the oxidized category, O/PVA exhibited a slightly smaller average pore size at 3.11 µm and a porosity of 2.81%. Moving on to filter papers enriched with both cationic starch and polyvinyl alcohol (U/CS/PVA and O/CS/PVA), we observed intriguing dynamics. U/CS/PVA and O/CS/PVA filter paper shared medium average pore sizes, with varying porosities of 4.06% and 4.64%, respectively. The results demonstrated that the responses of average pore size and porosity percentage were statistically significant. These results underscore the nuanced impact of additives on filter paper structures. The introduction of polyvinyl alcohol and the combined presence of cationic starch and polyvinyl alcohol appear to augment porosity while maintaining smaller pores due to the presence of a greater surface. This observation aligns with the findings of Ebrahimi et al. [41], indicating that oxidation of filter papers generally leads to increased permeability. Additionally, it aligns with the findings of Xu et al. [40], who observed a rough surface morphology with irregular pores in filter papers containing PVA. 

### 3.8. Fourier Transform Infrared Spectroscopy (FTIR)

**Figure 5:**
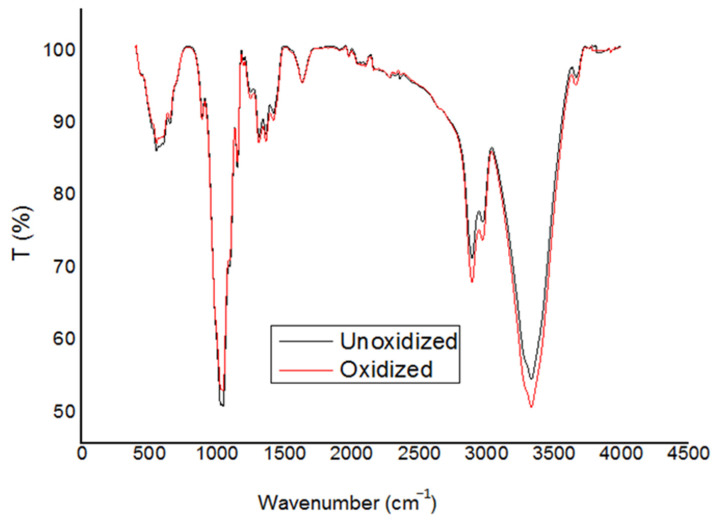
Infrared analysis of oxidized cellulose.

Figure 5 illustrates FTIR spectra of cellulose before and after oxidation treatment. The increase in intensity of the peak that can be observed at 3400–3500 corresponds to different O-H functional groups [42]. Additionally, the absorbance at approximately 1740 cm^−1^ is attributed to the C=O stretching vibration of carboxyl groups. This increase is due to the conversion of many hydroxyl groups of D-glucose units into carboxyl groups [43]. 

**Figure 6:**
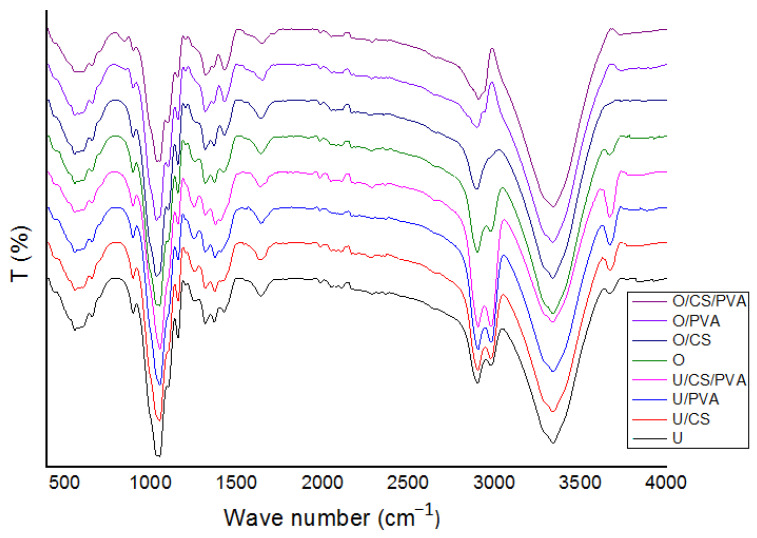
FTIR spectra (4000–400 cm^−1^) of filter papers.

All FTIR spectra in Figure 6 show a large broad band at 3400–3200 cm^−1^, which is related to the stretching of O-H bonds in hydroxyl, phenol, and carboxyl groups [44]. There are specific peaks for PVA, where the peaks in the 2900–2940 cm^−1^ and 3264 cm^−1^ regions relate to stretching vibration of methylene groups (C-CH_2_) and stretching vibrations of –OH groups, respectively [45,46]. The observed vibrational band at 2840–3000 cm^−1^ refers to C-H stretching of alkyl groups [46]. The bands at 1076 cm^−1^ are attributable to cationic starch. The peak at 1076 cm^−1^ corresponds to C1-H bending [47].

### 3.9. Nitrogen Adsorption Isotherms

**Figure 7:**
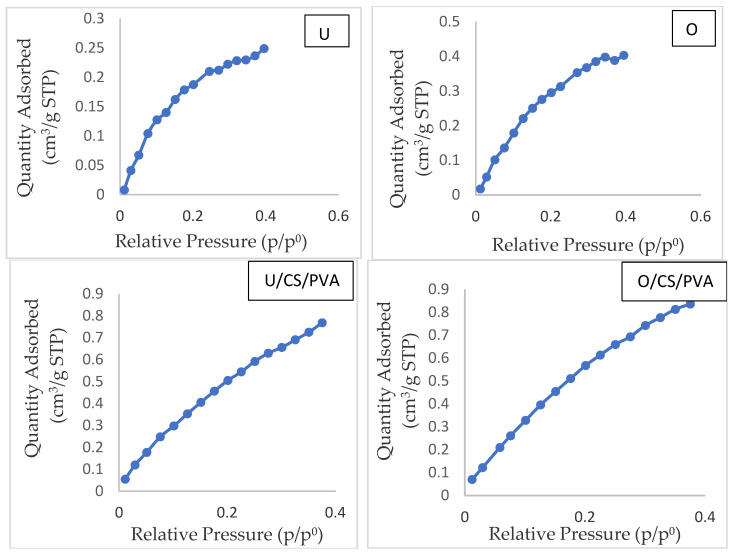
Adsorption–desorption isotherms of cellulose filter papers.

Figure 7 displays our nitrogen adsorption–desorption analysis of filter papers and determination of the specific surface area of the filter papers. The specific surface areas of the U, O, U/CS/PVA, and O/CS/PVA filter papers were determined to be 0.88, 1.35, 2.84, and 3.43 m^2^/g, respectively. Notably, the surface area in oxidized filters was greater than in unoxidized ones, and the addition of additives further increased the surface area. O/CS/PVA exhibited the highest specific surface area, while U exhibited the lowest. In the unoxidized sample, the maximum absorption value was 0.24 cm^3^/g, and with the reduction in the pressure ratio, the absorption value was at first approximately constant or decreased slowly, and then with a further decrease in the pressure ratio, the rate of reduction in the absorption value increased, while in the oxidized sample without additives, the maximum amount of absorption was 0.4 cm^3^/g, and with the reduction in the pressure ratio, the amount of absorption also decreased. In samples with additives, the amount of absorption reached 0.8 cm^3^/g, and the amount of absorption also decreased with the reduction in the pressure ratio. These results indicate that oxidation treatment and additives increase porosity while maintaining the size of the pores. These findings are consistent with the research of Shrotri et al. [48], which suggests that oxidation of filter papers increases porosity, thus increasing the surface area. Additionally, research by Azhar et al. [49] indicates that PVA creates pores in the composition, further enhancing porosity.

## 4. Conclusions

This study has explored the potential of bamboo fibers as a sustainable raw material for filter paper production without synthetic fibers and with appropriate mechanical resistance. Mechanical properties such as tear strength, burst resistance, and tensile strength were examined, and oxidized filters showed a significant effect in increasing these properties, especially when combined with PVA and CS. Air permeability measurements demonstrated nuanced effects of additives on porosity, and oxidized filters with the combined additives CS and PVA produced the highest air permeability. The combined addition of CS and PVA not only provided high mechanical strengths but also increased porosity while maintaining pore size. The results demonstrated that bamboo fibers, particularly when subjected to oxidation and specific additives, offer promise for sustainable filter paper production, providing a tailored approach to meeting diverse filtration needs. 

## Figures and Tables

**Table 1:** Sample identification showed additives and treatments.

Samples	Additives	Treatment
U	-	
U/CS	Cationic starch	
U/PVA	PVA	Unoxidized
U/CS/PVA	Cationic starch + PVA	
O	-	
O/CS	Cationic starch	Oxidized
O/PVA	PVA	
O/CS/PVA	Cationic starch + PVA	

**Table 2:** The yields, kappa numbers, and lignin percentages of the pulps.

Temperature (°C)	AQ (% Dry Weight)	Total Yield (%)	Kappa Number	Lignin (%)
185	0	28	26.10	4.3
185	0.1	31	24.66	4
185	0.2	35	20.54	3.38
175	0	35	46.46	7.6
175	0.1	38	36.45	6
175	0.2	42	17.5	2.8

**Table 3:** Air permeance of filter papers.

Filter Papers	Air Permeability (μm/Pa·s)	Filter Papers	Air Permeability (μm/Pa·s)
U	78.4 ± 1.07 *	O	79.1 ± 0.42
U/CS	77.3 ± 1.25	O/CS	78.4 ± 1.00
U/PVA	80.2 ± 1.52	O/PVA	79.4 ± 1.49
U/CS/PVA	81.35 ± 0.77	O/CS/PVA	83.8 ± 0.65

* Values are expressed as mean ± standard deviation.

**Table 4:** Pore sizes and porosities of filter papers, determined using ImageJ analysis of SEM images.

Filter Papers	Average Pore Size (µm)	Porosity (%)	Filter Papers	Average Pore Size (µm)	Porosity (%)
U	4.51	2.07	O	3.75	2.17
U/CS	1.44	0.66	O/CS	1.48	1.11
U/PVA	3.56	3.39	O/PVA	3.11	2.81
U/CS/PVA	3.90	4.06	O/CS/PVA	3.62	4.64

## Data Availability

Data are contained within the article.
